# Value of Half-Day Outpatient Management of Gestational Diabetes Mellitus Patients During the COVID-19 Epidemic

**DOI:** 10.1177/26884844251380118

**Published:** 2025-09-16

**Authors:** Weifu Song, Yanyan Wang, Fei Xiao

**Affiliations:** Department of Gynecology, Qinhuangdao Maternal and Child Health Hospital, Qinhuangdao, China.

**Keywords:** COVID-19 epidemic, gestational diabetes mellitus, half-day outpatient management

## Abstract

**Objective:**

To investigate the effects of half-day outpatient management of gestational diabetes mellitus (GDM) on blood glucose, fetal weight, maternal, and infant outcomes during the COVID-19 epidemic.

**Materials and Methods:**

From January 1, 2020, to December 31, 2022, 4674 pregnant women were diagnosed with GDM in the Woman and Child Care Center of Qinhuangdao City. Patients with GDM were divided into the case group and the control group according to their own wishes. To retrospectively analyze the differences in the maternal and infant outcomes between the two groups, the glucose tolerance, blood glucose levels before meals, 2 hours after meals, and at bedtime before delivery, hemoglobin A1C before delivery, mode of delivery, birth weight, and maternal and infant complications, perinatal complications of the two groups were observed.

**Results:**

There were significant differences in fasting blood glucose, blood glucose levels at 1 hour and 2 hours after sugar intake between the two groups at the time of admission. Before meals, 2 hours after meals, and at bedtime before delivery, blood glucose levels, and hemoglobin A1C were lower than those of the control group, and the difference was significant. There was no significant difference between the two groups in the complications of low-weight infants, neonatal asphyxia, stillbirth, polyamniotic fluid, premature rupture of membranes, hypertensive diseases during pregnancy, and postpartum hemorrhage. Significant differences were observed in complications such as macrosomia, neonatal hypoglycemia, and neonatal hyperbilirubinemia.

**Conclusions:**

The half-day outpatient management of GDM can effectively control the blood glucose level of pregnant women with GDM and improve clinical outcomes.

Gestational diabetes mellitus (GDM) is a form of diabetes that develops only during pregnancy, with normal glucose metabolism being observed before pregnancy. In recent years, there has been a significant global rise in the incidence of obesity and excessive weight gain during pregnancy. GDM poses serious hazards to both women and fetuses. GDM has significant short-term and long-term health risks for mothers, fetuses, and children. For mothers, complications such as gestational hypertension, excessive amniotic fluid, premature rupture of membranes, infection, and premature delivery are likely to occur. In severe cases, ketoacidosis may occur, and parturients may have postpartum diabetes for a long time.^1–3^ With the significant progress in recent years, the metabolic processes occurring during pregnancy and their effects on the intrauterine fetal development have been clearly defined.^4^ The American Diabetes Association updated the diagnostic criteria for GDM in 2024,^5^ leading to a notable increase in its reported incidence. With the liberalization of China’s “two-child” and “three-child” policies, there has been a gradual increase in the number of older pregnant women, contributing to a rise in the prevalence of GDM. The short-term risks for fetuses include macrosomia (excessive birth weight), shoulder dystocia, birth trauma, and postpartum hypoglycemia. For the offspring born to mothers with gestational diabetes, the long-term risks include an increased risk of obesity in childhood and adulthood, as well as an increased risk of cardiometabolic problems.^6^ To address these challenges, it is crucial to actively prevent GDM, identify high-risk pregnant women for GDM, and establish a safe and effective model for managing GDM during pregnancy. Dietary adjustments and increased physical activity are the main treatment methods for GDM, but when blood glucose does not reach normal levels, medications such as insulin are usually used for treatment. Some countries also use oral hypoglycemic drugs, mainly metformin and glibenclamide (glyburide). The treatment can improve immediate pregnancy outcomes, reducing fetal excessive growth, obesity, and pregnancy-related hypertensive diseases.^7^ Our hospital officially launched the one-day GDM outpatient service in 2013.

However, since the outbreak of COVID-19 in China at the end of 2019, in order to reduce exposure to the epidemic and ensure the management of patients with GDM during pregnancy, our hospital has changed the original one-day GDM outpatient management mode to the half-day outpatient management mode. Through the intervention of pregnant women with GDM in the half-day outpatient department and the traditional outpatient education management mode, the study compared the blood glucose situation of pregnant women in the two groups after intervention and the maternal and infant outcomes of the two groups, explored the value and influence of the GDM half-day outpatient department management mode on the pregnancy outcome of pregnant women with GDM, and provided scientific clinical intervention to guide pregnant women with GDM to control blood glucose scientifically, so as to improve the outcome of mother and child.

During the COVID-19 epidemic, the value of half-day outpatient management of patients with GDM is crucial for several reasons:

1. *Reducing exposure risk:* By choosing the half-day outpatient management, pregnant women with GDM can minimize their potential risk of COVID-19 infection in hospitals or healthcare facilities.
2. *Convenience and comfort:* Such management allows pregnant women to receive necessary care while still being able to return home the same day, thus ensuring their comfort and convenience.
3. *Effective monitoring:* Such management enables healthcare providers to regularly monitor the progress of patients with GDM in a timely manner, ensuring that any necessary adjustments to treatment plans can be made promptly.
4. *Resource optimization:* By utilizing such management, healthcare resources can be optimized, allowing for more efficient allocation of healthcare personnel and facilities during the epidemic.
5. *Patient empowerment:* Patients can be empowered through education and self-management techniques during half-day outpatient visits, leading to better health outcomes and improved understanding of their condition.

## Data and Methods

### General information

**FIG. 1.**
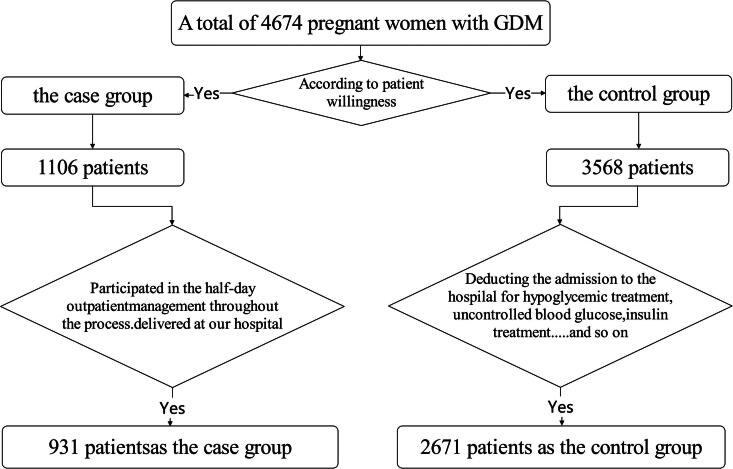
The flowchart of group screening.

Between January 1, 2020, and December 31, 2022, a total of 4674 pregnant women with GDM were admitted and delivered in our hospital. Pregnant women with GDM were divided into two groups according to their willingness to participate in the half-day diabetes outpatient management mode; 1106 pregnant women participated in the half-day diabetes outpatient management mode, with 931 giving birth in the hospital, selected as the case group. Among the remaining 3568 patients, after deducting the admission to the hospital for hypoglycemic treatment, uncontrolled blood glucose, insulin treatment, transfer of GDM experts, twin pregnancy, and delivery in other hospitals; 2671 patients were selected to adopt the traditional outpatient education management model and included in the control group. Figure 1 shows the flowchart of group screening for the case group and the control group.

*Inclusion criteria:* (1) The GDM diagnostic criteria in the Guidelines for Diagnosis and Treatment of Gestational Diabetes Mellitus (2014): for all pregnant women who have not yet been diagnosed with PGDM or GDM, 75 g OGTT was administered at 24–28 weeks of gestation and at the first visit after 28 weeks. Before taking sugar, 1 and 2 hours after taking sugar, the blood glucose values of the three items should be lower than 5.1, 10.0, and 8.5 mmol/L, respectively. Any blood glucose value meeting or exceeding the above criteria is diagnosed with GDM.^8^ (2) Regular obstetric examinations were conducted at the hospital throughout pregnancy and delivery. (3) Voluntary participation in diabetes management.

*Exclusion criteria:* (1) Preexisting diabetes or a history of GDM. (2) Other pregnancy complications. (3) Mental or cognitive disabilities. (4) Twin pregnancies. (5) Delivery in another hospital.

### Research methods

#### Intervention methods

**FIG. 2.**
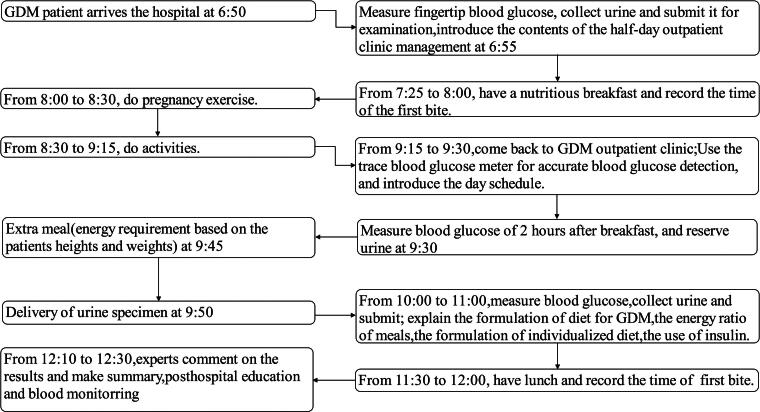
Half-day outpatient flowchart.

The management methods of the GDM half-day clinic for the research group are as follows: Make an appointment at the half-day clinic of our hospital every Wednesday. Inform pregnant women to have light food the night before, fast after 22:00, and avoid drinking water. Figure 2 presents the specific half-day outpatient flowchart.

The traditional outpatient education management mode for GDM pregnant women in the control group was carried out by obstetrical outpatient physicians and nurses. Based on the results of various examinations on the day, one-to-one teaching and educational guidance were provided on blood glucose detection, fetal movement count, reasonable diet, moderate exercise, medication methods, and precautions for pregnant women with GDM.

#### Evaluation indicators

The following aspects of pregnant women with GDM were compared between the two groups: blood glucose control and insulin application during pregnancy, delivery mode, and postpartum monitoring, including those with pregnancy-induced hypertension, premature rupture of membranes, excessive amniotic fluid, postpartum hemorrhage, macrosomia, full-term low body weight infants, neonatal hypoglycemia, neonatal asphyxia, and stillbirth.

##### Evaluation criteria:

(1) The criteria for blood glucose control were as follows: FPG ≤5.3 mmol/L, PBG ≤7.8 mmol/L at 1 hour, PBG ≤6.7 mmol/L at 2 hours, and blood glucose at night was no less than 3.3 mmol/L. The glycated hemoglobin (HbA1c) during pregnancy was <5.5%. After diet and exercise management, if the blood glucose failed to reach the above standards, insulin was added to the treatment as advised by the doctor.
(2) The diagnostic criteria for neonatal hypoglycemia are that blood glucose is ≤2.2 mmol/L.

### Statistical methods

SPSS 16.0 statistical analysis software was employed for data processing. A significance level of *p* < 0.05 indicated a statistically significant difference. The mean and standard deviation (X ± SD) were utilized for measurement data. The *t-test* and the rank sum test were employed to compare two independent samples, and the chi square test was applied for counting data.

## Results

### Comparison of detailed information about patients

Detailed information (average age, mean gestational time, average gestational age, systolic blood pressure, and diastolic blood pressure before and after the implementation of the intervention, body mass index) regarding the members of the case group and the control group is presented in Table 1. With the exception of the average gestational age, no statistically significant difference was identified between the case group and the control group (*p* > 0.05).

**Table 1.** Detailed Information between the Two Groups of Pregnant Women

No. of group	*N*	Average age/year	Mean gestational time/year	Systolic blood pressure before intervention/mmHg	Diastolic blood pressure before intervention/mmHg	Average gestational age/week	Systolic blood pressure/mmHg	Diastolic blood pressure/mmHg	Body mass index
Cases	931	31.7 ± 8.3	1.4 ± 0.5	109.2 ± 8.7	70.9 ± 2.7	38.9 ± 3.2	109.8 ± 7.2	75.5 ± 4.4	23.2 ± 3.1
Controls	2671	31.6 ± 8.1	1.4 ± 0.5	110.1 ± 10.0	70.9 ± 2.9	38.4 ± 3.6	109.5 ± 7.2	75.5 ± 4.3	23.2 ± 3.2
*T* value	—	0.441	0.551	−2.581	−0.308	3.582	1.224	0.223	0.336
*p* value	—	0.660	0.581	0.010	0.758	<0.001	0.221	0.824	0.737

Data (except for systolic blood pressure and diastolic blood pressure before intervention) in Table 1 are obtained from the period ranging from admission to the hospital to before delivery (1–2 days before delivery).

### Comparison of glucose tolerance

There were significant differences in fasting blood glucose, blood glucose levels at 1 hour and 2 hours after sugar intake, HbA1c between the two groups (*p* < 0.05). Before the blood glucose intervention was implemented, the glucose tolerance of the case group was higher than that of the control group, as illustrated in Table 2. Before the intervention, the blood glucose level in the case group was significantly higher (*p* < 0.05), which may be related to the patients in this group actively choosing a more intensive management mode. In the subsequent analysis, the baseline blood glucose level has been adjusted by covariance, and the difference in intervention effects between the two groups remained significant after adjustment (*p* < 0.05).

**Table 2.** Comparison of Glucose Tolerance between the Two Groups of Pregnant Women (mmol/L)

No. of group	*N*	Fasting blood glucose	Glucose after 1 hour	Glucose after 2 hours	Glycated hemoglobin
Cases	931	5.30 ± 0.58	10.04 ± 1.79	8.93 ± 1.98	6.05 ± 0.59
Controls	2671	5.17 ± 0.46	9.42 ± 1.62	8.40 ± 1.95	6.00 ± 0.55
*T* value	—	7.233	9.803	7.087	2.438
*p* value	—	<0.001	<0.001	<0.001	0.015

Data in Table 2 are obtained from before the implementation of the intervention.

Short-term follow-up results: At 6 weeks postpartum follow-up, the proportion of parturients with normalized fasting blood glucose in the case group was 89.4% (with 124 out of 139 actually followed-up having normal blood glucose levels), which was higher than that in the control group (76.3%, with 208 out of 273 actually followed-up having normal blood glucose levels, *p* < 0.05).

### Comparison of blood glucose before delivery

The blood glucose levels before breakfast, after meals, and at specific time points were significantly lower in the case group than in the control group (*p* < 0.05), suggesting better glucose control in the former group. The details are presented in Table 3.

**Table 3.** Comparison of Blood Glucose Levels before Three Meals, 2 Hours after Three Meals, Bedtime Blood Glucose, and Glycated Hemoglobin (mmol/L) between the Two Groups

No. of group	*N*	Before breakfast	2 hours after breakfast	Before lunch	2 hours after lunch	Before dinner	2 hours after dinner	Bedtime (22:00)	Glycated hemoglobin
Cases	931	5.14 ± 0.73	5.91 ± 1.04	4.80 ± 0.59	6.36 ± 1.20	5.19 ± 0.82	6.43 ± 1.42	4.76 ± 0.96	5.36 ± 0.70
Controls	2671	8.53 ± 2.65	9.11 ± 2.66	6.87 ± 1.22	7.18 ± 1.28	6.09 ± 1.00	7.37 ± 1.57	5.31 ± 1.03	5.56 ± 0.73
*T* value	—	−38.557	−35.739	−49.864	−17.167	−24.937	−16.094	−14.131	−7.394
*p* value	—	<0.001	<0.001	<0.001	<0.001	<0.001	<0.001	<0.001	<0.001

Data in Table 3 are obtained from the period ranging from admission to the hospital to before delivery (1–2 days before delivery).

### Comparison of pregnancy-related complications

The incidence of pregnancy-related complications, such as hypertension, premature rupture of membranes, excessive amniotic fluid, and postpartum hemorrhage, did not exhibit significant differences between the case group and the control group (*p* > 0.05), as indicated in Table 4.

**Table 4.** Comparison of Pregnancy Complications between the Two Groups

No. of group	*N*	Hypertensive disorders during pregnancy	Premature rupture of membranes	Hyperamniotic fluid	Postpartum hemorrhage
Cases	931	52 (5.6%)	31 (3.4%)	16 (1.7%)	7 (0.8%)
Controls	2671	126 (4.7%)	92 (3.5%)	67 (2.5%)	27 (1.0%)
Chi square	—	1.212	0.014	1.913	0.495
*p* value	—	0.156 (>0.05)	0.502 (>0.05)	0.102 (>0.05)	0.315 (>0.05)

### Comparison of perinatal complications

Significant differences were noted in neonatal complications such as macrosomia, neonatal hypoglycemia, and neonatal hyperbilirubinemia (*p* < 0.05) between the case group and the control group. Nevertheless, there were no significant disparities regarding low birth weight infants, neonatal asphyxia, and stillbirth (*p* > 0.05), as delineated in Table 5.

**Table 5.** Comparison of Perinatal Complications between the Two Groups

No. of group	*N*	Macrosomia	Low weight infants	Neonatal hypoglycemia	Neonatal asphyxia	Stillbirth	Neonatal hyperbilirubinemia	Preterm birth
Cases	931	70 (7.6%)	41 (4.4%)	72 (7.7%)	14 (1.5%)	0 (0.0%)	111 (12.0%)	30 (3.2%)
Controls	2671	257 (9.6%)	133 (5.0%)	266 (9.9%)	39 (1.5%)	2 (0.1%)	390 (14.6%)	136 (5.1%)
Chi square	—	3.539	0.528	3.865	0.004	0.698	3.877	2.577
*p* value	—	0.033 (<0.05)	0.264 (>0.05)	0.027 (<0.05)	0.527 (>0.05)	0.550 (>0.05)	0.027 (<0.05)	0.021 (<0.05)

### Comparison of delivery methods

The rates of vaginal delivery, cesarean section, and midwifery did not show statistically significant differences between the case group and the control group (*p* > 0.05), as presented in Table 6.

**Table 6.** Comparison of Delivery Modes between the Two Groups

No. of group	*N*	Eutocia	Cesarean section	Birth assistance (forceps/induction)
Cases	931	488 (52.4%)	420 (45.1%)	23 (2.5%)
Controls	2671	1447 (54.2%)	1168 (43.7%)	56 (2.1%)
χ^2^	1.137			
*p* value	0.566			

Overall, the study highlighted the positive impact of the GDM half-day clinic management mode on glucose control, maternal complications, and perinatal outcomes compared with the traditional outpatient education management mode.

## Discussion

Since the diagnostic criteria for gestational diabetes were proposed by O’Sullivan and Mahan in 1964, these criteria have continuously evolved. Although the original purpose of such criteria was mainly to assess the risk of mothers developing type 2 diabetes (T2D), subsequent studies were aimed at analyzing and quantifying the probability of adverse pregnancy and offspring outcomes.^9,10^ GDM poses risks to both mothers and offspring, influencing the entire pregnancy process. The short-term hazards for mothers encompass complications such as excessive amniotic fluid, secondary infections, and postpartum hemorrhage, while the long-term risk involves an increased likelihood of developing T2D.^11^ For the offspring, short-term hazards include preterm birth, malformation, fetal macrosomia, fetal growth restriction, fetal distress, postnatal hypoglycemia, and hyperbilirubinemia, which may heighten the risk of metabolic syndrome such as diabetes, hypertension, obesity, and coronary heart disease during adolescence or adulthood,^12^ and increasing evidence suggests GDM has significant short- and long-term complications for both the mother and the offspring.^6^ In 2010, with the support of the World Diabetes Fund, China carried out the “Cooperation Project for the Promotion of Standardized Diagnosis and Treatment of Gestational Diabetes Mellitus,”^13^ which gradually advanced the standardization process of clinical diagnosis and management of GDM in China, causing people to pay increasingly more attention to GDM.

In May 2011, the First Hospital of Peking University initiated the management mode of GDM one-day outpatient service,^14^ which achieved excellent results and has been widely promoted. Our hospital adopted a similar model in 2013, with customized schedules concentrating on education, meal planning, exercise, and glucose monitoring.

As pregnant women’s awareness of gestational diabetes has been increasing year by year, the number of GDM outpatient visits in our hospital has gradually risen. However, due to the COVID-19 outbreak, our hospital shifted to a half-day outpatient management mode, which also yielded promising results in this study. This study conducted a comparison of clinical outcomes between a case group and a control group of pregnant women. Apart from the average gestational age, no significant differences were observed in detailed patient information, including age, gestational time, blood pressure, and body mass index between the two groups (*p* > 0.05). The average gestational age in the case group was slightly longer (*p* < 0.001), which may be related to the reduced risk of preterm birth due to better blood glucose control (preterm birth rate: 3.2% in the case group vs. 5.1% in the control group, *p* = 0.021).

Significant disparities in fasting blood glucose and blood glucose levels at 1 and 2 hours after sugar intake were identified between the groups (*p* < 0.05), with the case group showing higher glucose tolerance before the intervention. The case group exhibited significantly lower blood glucose levels before and after meals compared with the control group (*p* < 0.05). No significant differences were found in pregnancy-related complications such as hypertension, premature rupture of membranes, excessive amniotic fluid, and postpartum hemorrhage between the groups (*p* > 0.05). However, significant differences were noted in perinatal complications such as macrosomia, neonatal hypoglycemia, and neonatal hyperbilirubinemia (*p* < 0.05), while no significant differences were observed in low birth weight infants, neonatal asphyxia, and stillbirth (*p* > 0.05). Additionally, there were no statistically significant differences in delivery methods between the two groups (*p* > 0.05). Overall, the GDM half-day clinic management mode had positive impacts on glucose control, maternal complications, and perinatal outcomes compared with the traditional outpatient education management mode.

The increased awareness of GDM has led to a rise in outpatient visits, and the shift to half-day management during the epidemic has produced positive results. The study’s findings emphasize the significance of tailored management approaches, such as dietary guidance and exercise control, in improving outcomes for pregnant women with GDM. The study highlights the effectiveness of the GDM half-day outpatient management model in controlling blood glucose levels and enhancing the overall well-being of mothers and neonates.

Consistent with the findings of Eberle et al.’s (2021) research on remote GDM management during the pandemic,^15^ telemedical interventions have demonstrated positive effects on blood glucose control. By reducing in-person contact to lower infection risks, telemedicine emerged as an effective alternative for GDM management amid the pandemic. Their study verified the improving effects of remote interventions (including web-based and app-based models) on HbA1c and fasting blood glucose, providing a feasible approach for GDM management during special periods. Meanwhile, the half-day outpatient model adopted in this study shows superior efficacy in blood glucose control. Considering the characteristics of both models, the advantages of telemedicine in reducing the number of medical visits and enhancing convenience complement the strengths of the half-day outpatient model in improving the precision of blood glucose control. Therefore, future research could explore a hybrid management model of “half-day outpatient + remote follow-up,” which would not only achieve precise interventions through centralized outpatient services but also maintain the continuity of long-term management *via* remote follow-up. This would ensure effective blood glucose control while addressing the needs of pandemic prevention and control as well as the convenience of patients’ medical visits.

### Limitations of this study

In the grouping process, participants were not randomly assigned to the intervention group according to their own will. There may be some differences between the control group and the intervention group, which may affect the research results. In addition, there are still 175 patients who did not fully participate in the half-day outpatient management of gestational diabetes. There is a certain phenomenon of data missing, which may have some unknown impacts on the research results.

## Conclusion

The research on different diabetes education models has developed from traditional didactic teaching to interactive and collaborative health education approaches supported by theoretical foundations.^16^ Although various studies have documented the practical application of these models, there is still no universally recognized “best” education theory and method.^17^ In the context of this study, which employed a nonrandomized grouping approach, the results may be susceptible to selection bias. Consequently, future research should seek to further verify the effectiveness of the half-day outpatient management model through randomized controlled trials.

In China, the current treatment method for GDM conforms to the “five carriage” theory of diabetes treatment, highlighting health education, medical nutrition therapy, exercise therapy, self-glucose monitoring, and insulin therapy. The development and implementation of the GDM half-day outpatient management mode have demonstrated positive effects in controlling blood glucose levels and managing maternal and infant complications effectively. Through the utilization of this innovative management model, the majority of pregnant women with GDM can keep their blood sugar within normal ranges without the need for hospitalization. This not only leads to cost savings in medical care but also provides convenience to pregnant women by offering comprehensive care and support. The GDM half-day outpatient management mode represents a considerable advancement in the management of gestational diabetes, enhancing the outcomes for both mothers and infants in China.

## Abbreviation Used

COVID-19 epidemic: Coronavirus Disease 2019 Epidemic
GDM: Gestational Diabetes Mellitus
